# Arthroscopic microfracture with atelocollagen augmentation for osteochondral lesion of the talus: a multicenter randomized controlled trial

**DOI:** 10.1186/s12891-020-03730-3

**Published:** 2020-11-03

**Authors:** Young Koo Lee, Ki Won Young, Jin Su Kim, Hong Seop Lee, Whi-Je Cho, Hyong Nyun Kim

**Affiliations:** 1 Department of Orthopedic Surgery, Soonchunhyang University Bucheon Hospital, Bucheon-si, Gyunggi-do Republic of Korea; 2 Department of Foot and Ankle Surgery, Eulji Medical Center, Eulji University, Seoul, Republic of Korea; 3 Department of Orthopedic Surgery, Sejong Sports Medicine and Performance Center, Seoul, Republic of Korea; 4 Department of Orthopedic Surgery, Kangnam Sacred Heart Hospital, Hallym University College of Medicine, 1, Singil-ro, Yeongdeungpo-gu, Seoul, 07441 Republic of Korea

**Keywords:** Ankle, Arthroscopy, Atelocollagen, Microfracture, Osteochondral lesion

## Abstract

**Background:**

We aimed to evaluate whether arthroscopic microfracture with atelocollagen augmentation could improve the clinical outcomes and quality of regenerated cartilage in patients with osteochondral lesion of the talus (OLT). We hypothesized that the clinical outcomes and quality of the regenerated cartilage would be superior in patients undergoing arthroscopic microfracture with atelocollagen augmentation compared to those undergoing arthroscopic microfracture alone.

**Methods:**

In this multicenter, randomized controlled trial, 60 patients were randomly allocated to two groups: arthroscopic microfracture with atelocollagen augmentation (group 1, *n* = 31) and arthroscopic microfracture alone (group 2, *n* = 29). Mean 100-mm visual analog scale (VAS), Hannover scoring system (HSS), and American Orthopedic Foot and Ankle Society (AOFAS) scores were assessed 2 years postoperatively and compared between the groups. The quality of the regenerated cartilage was assessed according to the Magnetic Resonance Observation of CArtilage Repair Tissue (MOCART) score based on magnetic resonance imaging.

**Results:**

Forty-six patients (22 in group 1, 23 in group 2) completed the 2-year follow-up. The quality of the regenerated cartilage assessed based on the MOCART score was significantly superior in group 1 compared to group 2 (64.49 ± 18.27 vs 53.01 ± 12.14, *p* = 0.018). Clinical outcomes in terms of 100-mm VAS (17.25 ± 20.31 vs 19.37 ± 18.58, *p* = 0.72), HSS (93.09 ± 13.64 vs 86.09 ± 13.36, *p* = 0.14), and AOFAS (91.23 ± 8.62 vs 86.91 ± 10.68, *p* = 0.09) scores were superior in group 1 compared to group 2, but the differences were not statistically significant. Both groups showed significant improvements in clinical outcomes compared with the preoperative values.

**Conclusion:**

The quality of the regenerated cartilage was superior after arthroscopic microfracture with atelocollagen augmentation compared to that after microfracture alone in patients with OLT. Clinical outcomes assessed 2 years postoperatively were superior in patients who underwent arthroscopic microfracture with atelocollagen augmentation compared to those who underwent arthroscopic microfracture alone, although the differences were not statistically significant. A long-term study of the cohort is required to confirm these findings.

**Trial registration:**

ClinicalTrials.gov (NCT02519881), August 11, 2015.

## Background

Arthroscopic microfracture is the most frequently performed procedure for an osteochondral lesion of the talus (OLT) [1–4]. Although the short- to mid-term clinical outcomes are generally good, the quality of regenerated cartilage is unpredictable. After a longer follow-up, some patients showed that the beneficial results were not maintained, indicating the deterioration of the regenerated cartilage [5, 6]. Arthroscopic microfracture has several limitations. The blood clot formed after the microfracture may not be sufficiently mechanically stable to withstand tangential forces and may be washed out by the synovial fluid [7]. Further, it can be damaged by axial forces and the regeneration abilities of the chondrocytes may disappear [8]. The damaged parts may be filled with fibrous cartilage instead of hyaline cartilage. Accordingly, many strategies have been introduced to improve the quality of the regenerated cartilage [9].

Atelocollagen, a highly purified cell-free type I collagen, has been developed to provide matrix stability and maintain blood clotting at the defect site [8, 10, 11]. Type I collagen is a major component of the extracellular matrix, which is an important constituent of articular cartilage. To eliminate the immune function of collagen, atelocollagen, which has the antigenic telopeptide removed at both ends of collagen’s triple helix structure, can be used. A mixture of atelocollagen and fibrin glue can be directly injected arthroscopically on the cartilage defect, which solidifies after polymerization on the site when the solid-type collagen matrix needs an open approach. This feature makes atelocollagen augmentation suitable for an ankle joint characterized by difficult surgical access that sometimes requires malleolar osteotomy to access the OLT. It is a one-step procedure that does not require harvesting of healthy cartilage or mesenchymal stem cell from the donor site. Satisfactory regeneration of cartilage and clinical outcome improvements have been demonstrated in several case series of OLT [8, 12, 13]. However, to the best of our knowledge, there has been no randomized control trial on the application of this technique for OLT.

The purpose of this multicenter, randomized controlled trial was to evaluate whether arthroscopic microfracture with atelocollagen augmentation could improve the clinical outcomes and quality of regenerated cartilage in patients with OLT. We hypothesized that the clinical outcomes and quality of the regenerated cartilage would be superior in patients undergoing arthroscopic microfracture with atelocollagen augmentation compared to those undergoing arthroscopic microfracture alone.

## Materials and methods

This multicenter, randomized controlled trial assessed the clinical and radiological outcomes of patients who underwent arthroscopic surgery for OLT. Participants were enrolled from three university hospitals. The study was designed and implemented following the Consolidated Standards of Reporting Trials (CONSORT) statement and was approved by the Institutional Review Board of all the participating hospitals. This study was registered with ClinicalTrials.gov (NCT02519881). Written informed consent was obtained from all participants.

### Eligibility criteria

**Fig. 1:**
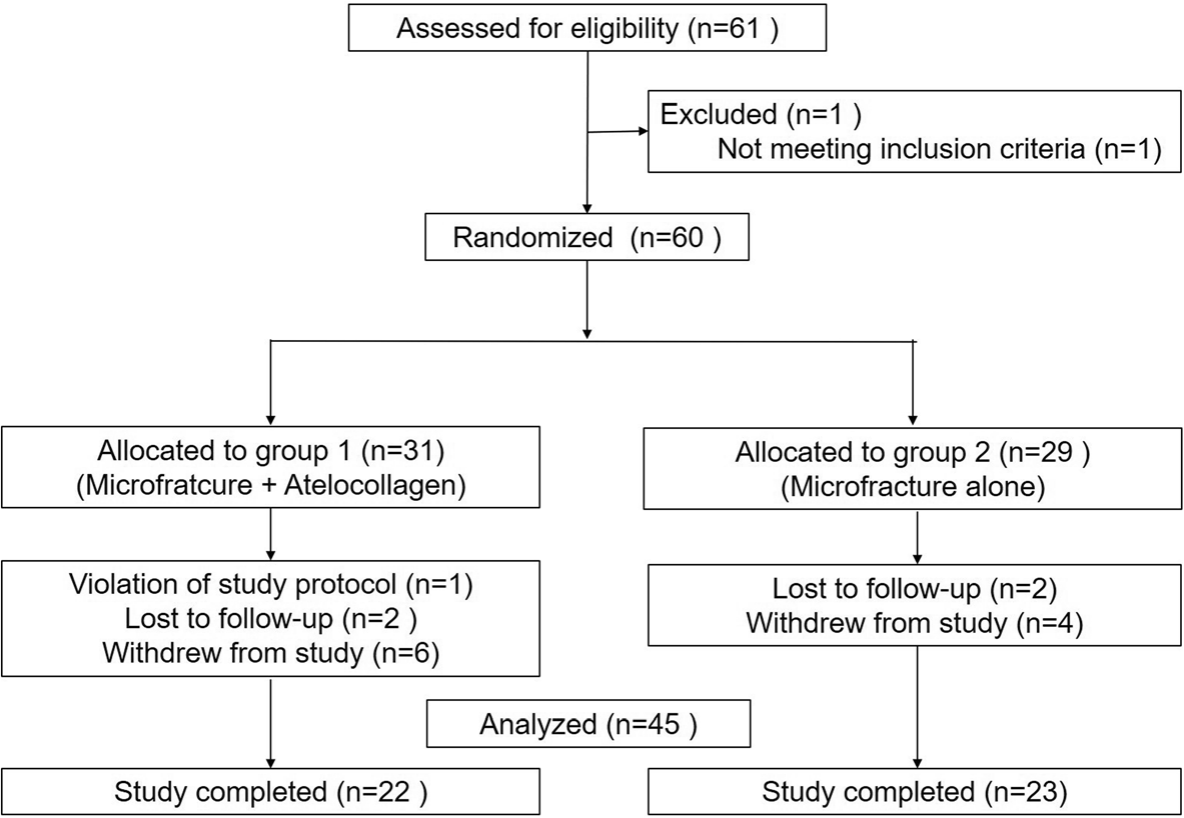
Consolidated Standards of Reporting Trials (CONSORT) flow diagram. Group 1 = experimental group (microfracture + atelocollagen augmentation), Group 2 = control group (microfracture alone)

Patients who were over 15 years of age with OLT requiring surgical treatment due to the failure of conservative treatment were prospectively enrolled in the study (Fig. 1). All patients were primary cases and were questioned on general health information to exclude patients with conditions that could potentially affect the healing process of the talus, including uncontrolled diabetes, autoimmune disease, a history of anaphylaxis, systemic inflammatory disease, or other conditions that would prevent them from following the study protocol.

### Randomization

Patients were randomly allocated to one of the two study groups: the experimental group treated with arthroscopic microfracture combined with atelocollagen augmentation (group 1) or the control group treated with arthroscopic microfracture alone (group 2). A computerized block randomization allocation method was used. A randomization list was generated using a 1:1 allocation and was stratified by the study centers. The investigators were blinded to ensure allocation concealment before the surgery.

### Preoperative evaluation

Preoperatively, all lesions were evaluated by plain ankle radiography and magnetic resonance imaging (MRI) to assess the size, location, shape, and morphology of the lesion. The defect size was defined and determined on MR images according to the method by Choi et al., whereby the area was calculated by the ellipse formula of coronal length×sagittal length× 0.79 [14, 15]. MRI was used to assess the condition of the cartilage overlying the osteochondral fragment.

### Surgical procedures

**Fig. 2:**
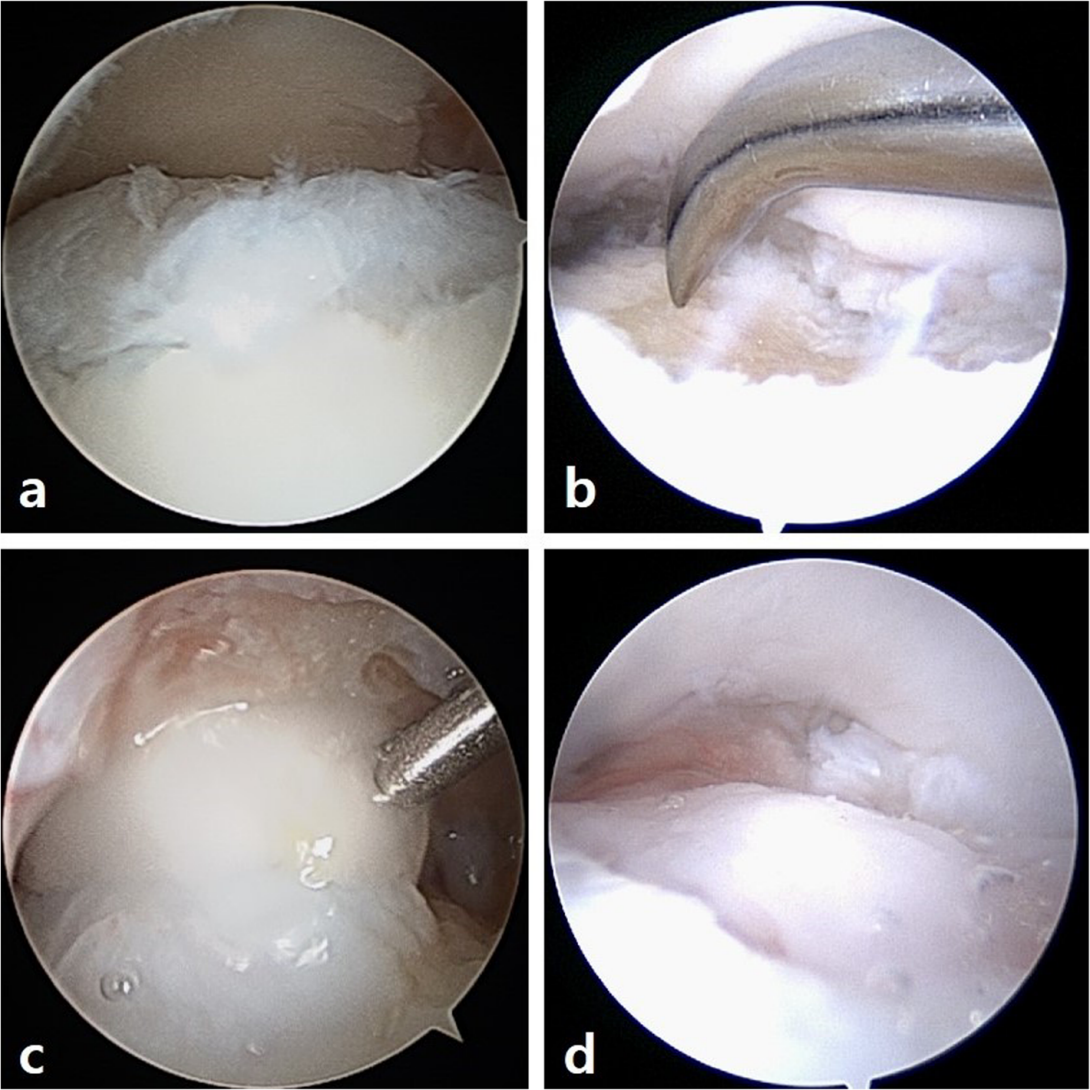
Arthroscopic images showing the steps of microfracture with atelocollagen augmentation. **a,** Chondral lesion of the talus was evaluated. **b**, Microfracture was performed. **c**, The gel, atelocollagen, and fibrin mixture was slowly applied to the defect. **d**, Stability was verified by repeated dorsiflexion and plantarflexion of the ankle joint

All surgical procedures were performed under general or spinal anesthesia. In both groups, ankle arthroscopy was performed through standard anteromedial and anterolateral portals. The lesion was evaluated and graded according to the International Cartilage Repair Society (ICRS) grading system [11, 16]. Patients underwent a standard arthroscopic microfracture as described previously for both groups [8, 17]. Fat droplets were checked after microfractures placed 3–4 mm apart. Then for group 1, intra-articular fluid of the ankle joint was removed by suction and atelocollagen augmentation was performed. A Y-shaped mixing catheter connected two 1-mL syringes, one filled with 0.9 mL of atelocollagen (CartiFill; Sewon Cellontech, Co., Ltd., Seoul, Korea) and 0.1 mL of thrombin (50 IU) and another filled with 1 mL of fibrin glue (Greenplast®, Green Cross PD. Co., Yongin, Korea). Under arthroscopic vision, the gel, in a two-way syringe, was mixed and slowly applied into the defect. An initial atelocollagen layer was generated. After 1 to 2 min, an additional layer was produced on the top of the initial layer to form a complete seal. Atelocollagen mixed with fibrinogen and thrombin could solidify and maintain the shape of the articular surface approximately 5 min after application because of the reaction between the thrombin and fibrinogen. Matrix stability was checked several times by dorsiflexion and plantarflexion of the ankle joint (Fig. 2). For group 2, microfracture was performed without atelocollagen augmentation. The arthroscopic portals were closed and a posterior splint was applied for 4 weeks before range of motion exercises started. Partial weightbearing was encouraged at 2 weeks postoperatively, and full weightbearing was permitted after 4 weeks. Patients were allowed to jog 3 months postoperatively.

### Second-look arthroscopic surgery and histologic evaluation

A second-look arthroscopic surgery was performed 2 years postoperatively for patients who had agreed to the surgery and a tissue biopsy. The regenerated cartilage was graded according to the ICRS grading system [11, 16]. One biopsy specimen was taken using a chondral biopsy needle. The biopsy samples were stained with hematoxylin and eosin, Masson’s trichrome for cell distribution, and collagen. Specimens were stained with Safarinin O, Alcin blue, and Toluidine blue for glucoaminoglycan distribution. The sections were also immunostained using collagen type-specific antibodies for type I collagen and type II collagen distribution. For histological assessment, the thickness of the cartilage, the Oswestry (Os) score, and immunohistochemistry scoring for type I and type II collagen were evaluated [18, 19]. Histological assessment was carried out by three independent observers, and the mean of three values was used as the final value.

### Outcome measures

The clinical outcome measures were assessed by the mean values of the 100-mm visual analog scale (VAS), Hannover Scoring System (HSS) for the ankle [8, 17], and the American Orthopedic Foot and Ankle Society (AOFAS) ankle hindfoot scores [20] assessed at the 2-year follow-up by an independent investigator who was blinded to the study. The 100-mm VAS is a validated self-assessment tool for evaluating pain after surgery [21–24]. Patients were asked to indicate their current pain severity with a single vertical mark through a 100-mm horizontal VAS bounded by the descriptors “least possible pain” at 0 mm and “worst possible pain” at 100 mm. The mean 100-mm VAS score was compared between the groups and the preoperative and postoperative values were compared to assess improvement after the treatment. The HSS incorporates clinical evaluation, functional performance, and subjective patient assessment. This ankle-specific outcome instrument has been previously used to assess clinical outcomes after cartilage repair [8, 17]. The quality of the regenerated cartilage was assessed according to the magnetic resonance observation of cartilage repair tissue (MOCART) score [25, 26]. The MOCART is a validated assessment tool used to perform structured morphological assessment of articular cartilage repair, with 100 set as the best possible score and 0 as the worst possible score [25, 26]. MRI was performed at the 2-year postoperative follow-up and two independent investigators (orthopedic surgeons), who were blinded to the study, evaluated the MOCART score twice with an interval of 2 weeks. The mean value of the two observers’ measurements was used. All adverse events and complications were evaluated and were recorded according to the principles of good clinical practice.

### Statistical analysis

The sample size was determined assuming 80% power and a 0.05 significance level. We performed a pilot study on 16 patients and assessed their postoperative 100-mm VAS scores to calculate the sample size required for the randomized controlled trial. With a pooled standard deviation of 14.56, a sample size of 48 patients was required to obtain a power of 80%. By assuming a drop-out rate of 20%, we selected a sample size of 60 patients for the present study.

Todd et al. reported that based on a study of 48 patients, the minimum clinically important difference (MCID) in 100-mm VAS score was 13 mm and that any differences below this amount, even if statistically significant, were unlikely to be of clinical significance [24]. Kelly et al. reported the MCID to be 9 mm [23]. We therefore set the MCID at 12 mm in this study. Data normality was assessed using the Kolmogorov–Smirnov test. Baseline patient characteristics and postoperative outcome measures were compared between the two groups using the independent t-test or the Mann-Whitney U test. The mean preoperative and postoperative outcome measures were compared using the paired t-test or the Wilcoxon signed-rank test. Differences in categorical variables were tested using the chi-squared test. Statistical significance was set at *p* <  0.05. All analyses were completed by a biostatistician with the use of SPSS version 22.0 (IBM Corporation, Armonk, NY).

## Results

A total of 61 patients were assessed for eligibility (Fig. 1). One patient who did not meet the inclusion criteria was excluded in the initial screening and 60 patients were included in the study. Of these, 31 patients were randomized to the experimental group (group 1) and 29 to the control group (group 2). The patients’ baseline characteristics were similar between the groups (Table 1).

**Table 1:** Demographic and Preoperative Data of Patients

	Group 1 (***n*** = 31)^a^	Group 2 (***n*** = 29)^a^	p-value
**Age (y)**	35.03 ± 15.69	39.10 ± 14.86	0.35
**Sex (male/female)**	15/16	13/16	0.78
**Height (cm)**	166.16 ± 9.64	164.45 ± 9.76	0.50
**Weight (kg)**	72.51 ± 17.25	75.54 ± 19.81	0.47
**BMI (kg/m**^**2**^**)**	26.14 ± 4.24	27.66 ± 4.85	0.31
**Lesion size (mm**^**2**^**)**	96.84 ± 71.93	109.43 ± 11.93	0.78
**ICRS grade**			
**Grade II, n (%)**	0 (0.00%)	2 (3.33%)	0.26
**Grade III, n (%)**	7 (11.67%)	9 (15.00%)	
**Grade IV, n (%)**	24 (40.00%)	18 (30.00%)	
**Preoperative 100-mm VAS**	51.37 ± 19.04	56.78 ± 19.73	0.10

^a^Values are given as the mean ± standard deviation, with the exception of sex and ICRS grade. *BMI* body mass index, *ICRS* international cartilage repair society, *VAS* visual analog scale. Group 1: experimental group (microfracture + atelocollagen augmentation), Group 2: control group (microfracture alone)

One patient violated the study protocol, two patients were lost to follow-up, and six patients in group 1 withdrew from the study. Two patients were lost to follow-up and four patients withdrew from the study in group 2. These 15 patients were excluded from the outcome analysis, leaving 22 and 23 patients in group 1 and group 2, respectively, for the analysis. The results are summarized in Tables 2, 3, 4 and 5. Clinical outcomes in terms of the mean 100-mm VAS (17.25 ± 20.31 vs 19.37 ± 18.58, *p* = 0.72), HSS (93.09 ± 13.64 vs 86.09 ± 13.36, *p* = 0.14), and AOFAS (91.23 ± 8.62 vs 86.91 ± 10.68, *p* = 0.09) scores assessed at 2 years postoperatively were superior in group 1 compared to group 2, but the differences were not statistically significant (Table 2).

**Table 2:** Clinical Outcome Measures

	Preoperative^a^	2-year follow-up^a^	Difference^†^	p-value^**‡**^	p-value^**§**^
**100-mm VAS**					
**Group 1**	51.84 ± 19.98	17.25 ± 20.31	−34.59 (95% CI, −47.61 to −21.57)	< 0.001	0.72
**Group 2**	59.11 ± 19.67	19.37 ± 18.58	− 39.74 (95% CI, − 49.83 to − 29.64)	< 0.001	
**HSS**					
**Group 1**	68.82 ± 11.86	93.09 ± 13.64	24.27 (95% CI, 16.49 to 32.06)	< 0.001	0.14
**Group 2**	67.57 ± 14.74	86.09 ± 13.36	18.52 (95% CI, 11.19 to 25.86)	< 0.001	
**AOFAS**					
**Group 1**	72.23 ± 11.85	91.23 ± 8.62	19.00 (95% CI, 11.97 to 26.03)	< 0.001	0.09
**Group 2**	69.30 ± 17.97	86.91 ± 10.68	17.61 (95% CI, 9.02 to 26.20)	< 0.001	

^a^The values are given as mean ± standard deviation. †The values are given as the mean and the 95% CI in parentheses. ‡p-values are calculated comparing the 2-year follow-up and preoperative values. §p-values are calculated comparing group 1 and group 2 values. Group 1 = experimental group (microfracture + atelocollagen augmentation), Group 2 = control group (microfracture alone), *VAS* visual analog scale, *HSS* Hannover scoring system, *CI* confidence interval; *AOFAS* American Orthopedic Foot and Ankle Society

**Table 3:** MOCART scores

Variables	Scores^a^	Group 1 (***n*** = 22)^b^	Group 2 (n = 22)^b^	p-value
**1. Degree of defect repair and filling of the defect**	20	14.89 ± 3.39	14.32 ± 4.40	0.63
**2. Integration of the border zone**	15	10.85 ± 3.69	9.83 ± 3.67	0.36
**3. Surface of the repair tissue**	10	7.73 ± 2.55	5.68 ± 2.31	0.008
**4. Structure of the repair tissue**	5	3.24 ± 1.99	2.27 ± 2.17	0.13
**5. Signal intensity of the repair tissue**	30	14.32 ± 8.83	8.01 ± 4.43	0.005
**6. Subchondral lamina**	5	2.95 ± 1.83	1.93 ± 2.17	0.10
**7. Subchondral bone**	5	2.16 ± 2.22	2.61 ± 2.14	0.49
**8. Adhesion**	5	4.55 ± 1.19	4.77 ± 0.63	0.43
**9. Effusion**	5	3.81 ± 2.02	3.58 ± 2.05	0.71
**Total Scores**	100	64.49 ± 18.27	53.01 ± 12.14	0.018

^a^The highest scores possible for each variable. ^b^Values are given as the mean ± standard deviation. MOCART = magnetic resonance observation of cartilage repair tissue, Group 1 = experimental group (microfracture + atelocollagen augmentation), Group 2 = control group (microfracture alone)

**Table 4:** Inter-observer and intra-observer reliability of MOCART score measurements

	MOCART		
	ICC	95% CI	p-value
Inter-observer reliability	0.844	0.695–0.918	< 0.001
Intra-observer reliability			
Observer 1	0.821	0.674–0.902	< 0.001
Observer 2	0.968	0.942–0.983	< 0.001

*MOCART* magnetic resonance observation of cartilage repair tissue, *ICC* intraclass correlation coefficient, *CI* confidence interval

**Table 5:** ICRS Grades at Second-look Arthroscopy

Grades	Group 1 (***n*** = 6)^a^	Group 2 (n = 2)^a^
**Grade 0**	2 (25%)	0 (0%)
**Grade I**	5 (37.5%)	2 (25%)
**Grade II**	1 (12.5%)	0 (0%)
**Grade III**	0 (0%)	0 (0%)
**Grade IV**	0 (0%)	0 (0%)

^a^Values are given as the number of the patients with percentage in parenthesis. Group 1 = experimental group (microfracture + atelocollagen augmentation), Group 2 = control group (microfracture alone), ICRS=International Cartilage Repair Society

Patients in both groups showed significant improvement in the mean 100-mm VAS, HSS, and AOFAS scores when compared to the pre- and postoperative score values (Table 2). The mean 100-mm VAS score significantly improved from a preoperative mean of 51.84 ± 19.98 to a postoperative mean of 17.25 ± 20.31 (*p* <  0.001) in group 1. The improvement of 34.59 ± 29.36 (95% confidence interval [CI], 21.57 to 47.61) was greater than the MCID of 12 mm. The mean 100-mm VAS score also significantly improved from a preoperative mean of 59.11 ± 19.67 to a postoperative mean of 19.37 ± 18.58 (p <  0.001) in group 2. The improvement of 39.74 ± 23.34 (95% CI, 29.64 to 49.83) mm was greater than the MCID of 12 mm (Table 3). More patients were pain-free during physical activity in group 1 than in group 2 [11 [50%] vs 5 [22%], *p* = 0.048). The mean pain score during physical activity (none = 5, yes = 0) was better for group 1 (2.50 ± 2.56) than for group 2 (1.09 ± 2.11), although the difference showed a borderline level of statistical significance (*p* = 0.0519). The mean MOCART score at the 2-year follow-up was significantly higher in group 1 than in group 2 (64.49 ± 18.27 vs 53.01 ± 12.14, *p* = 0.018) (Table 3).

**Fig. 3:**
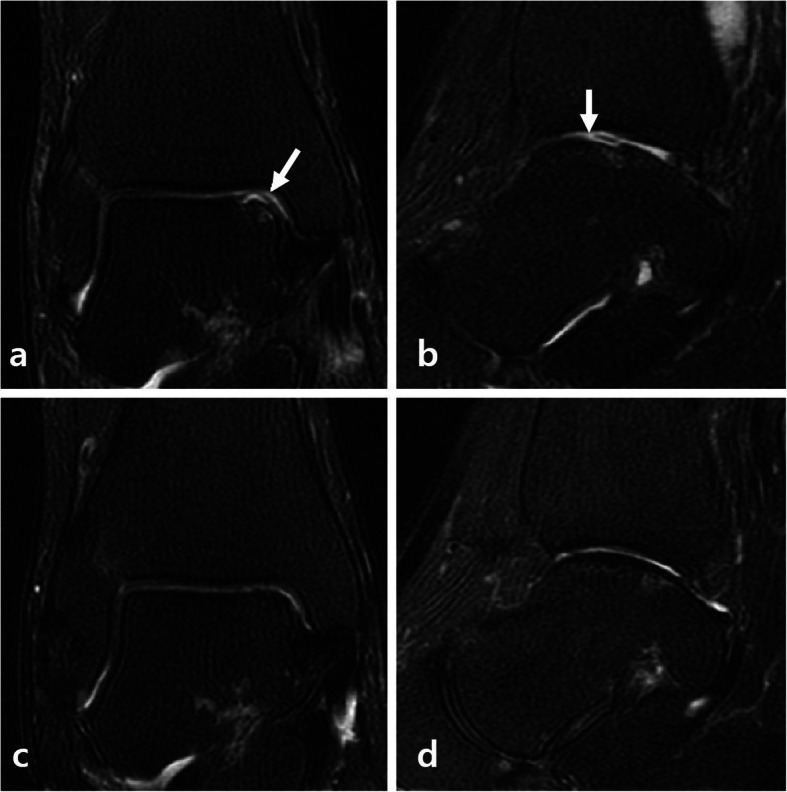
Preoperative magnetic resonance imaging (MRI) showing (**a-b**) an osteochondral lesion of the talus (arrow). **c-d,** Complete filling of the osteochondral defect 2 years after arthroscopic microfracture with atelocollagen augmentation

MRI outcomes showed that the surface of the repair tissue was significantly superior in group 1 compared to group 2 (7.73 ± 2.55 vs 5.68 ± 2.31, *p* = 0.008) (Fig. 3). The signal intensity of the repair tissue was significantly higher in group 1 than in group 2 (14.32 ± 8.83 vs 8.01 ± 4.43, *p* = 0.005). The intra- and inter-observer reliability of the measurement of the MOCART score were acceptable (Table 4).

However, there was no correlation between the MOCART score and any of the clinical outcome scores (*p* = 0.55, *p* = 0.88, and *p* = 0.78 for the 100-mm VAS, HSS, and AOFAS scores, respectively) [27].

**Fig. 4:**
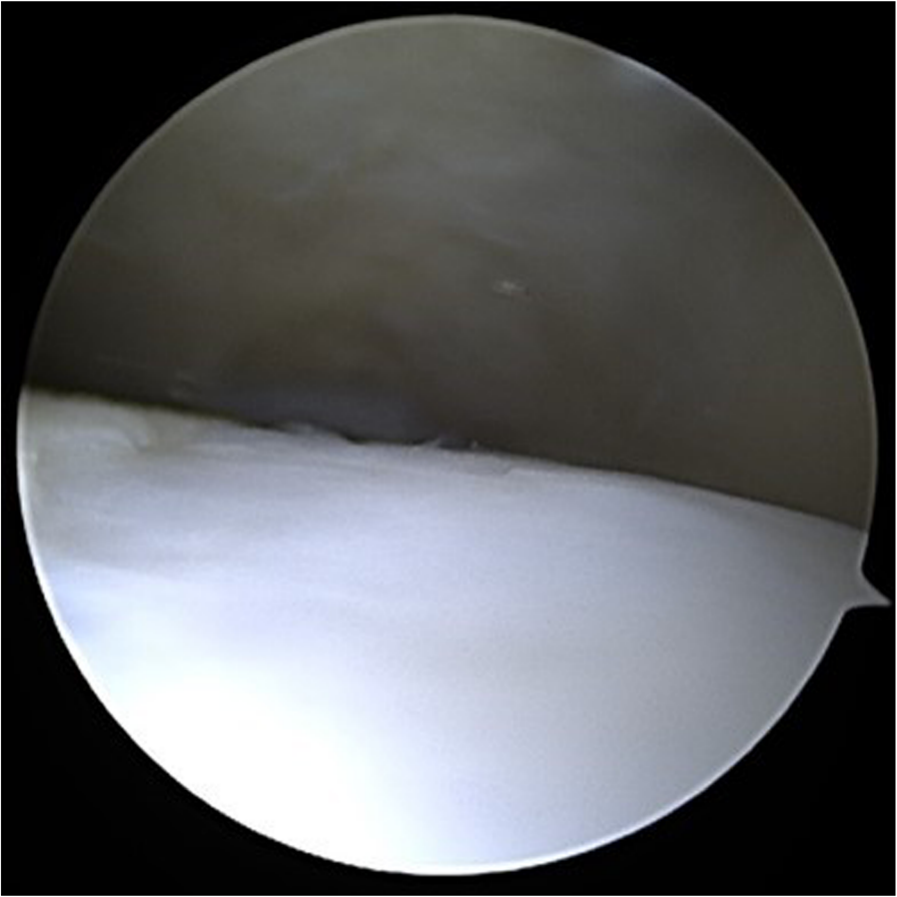
Excellent cartilage repair was seen in the second-look arthroscopy performed 2 years after microfracture with atelocollagen augmentation

**Fig. 5:**
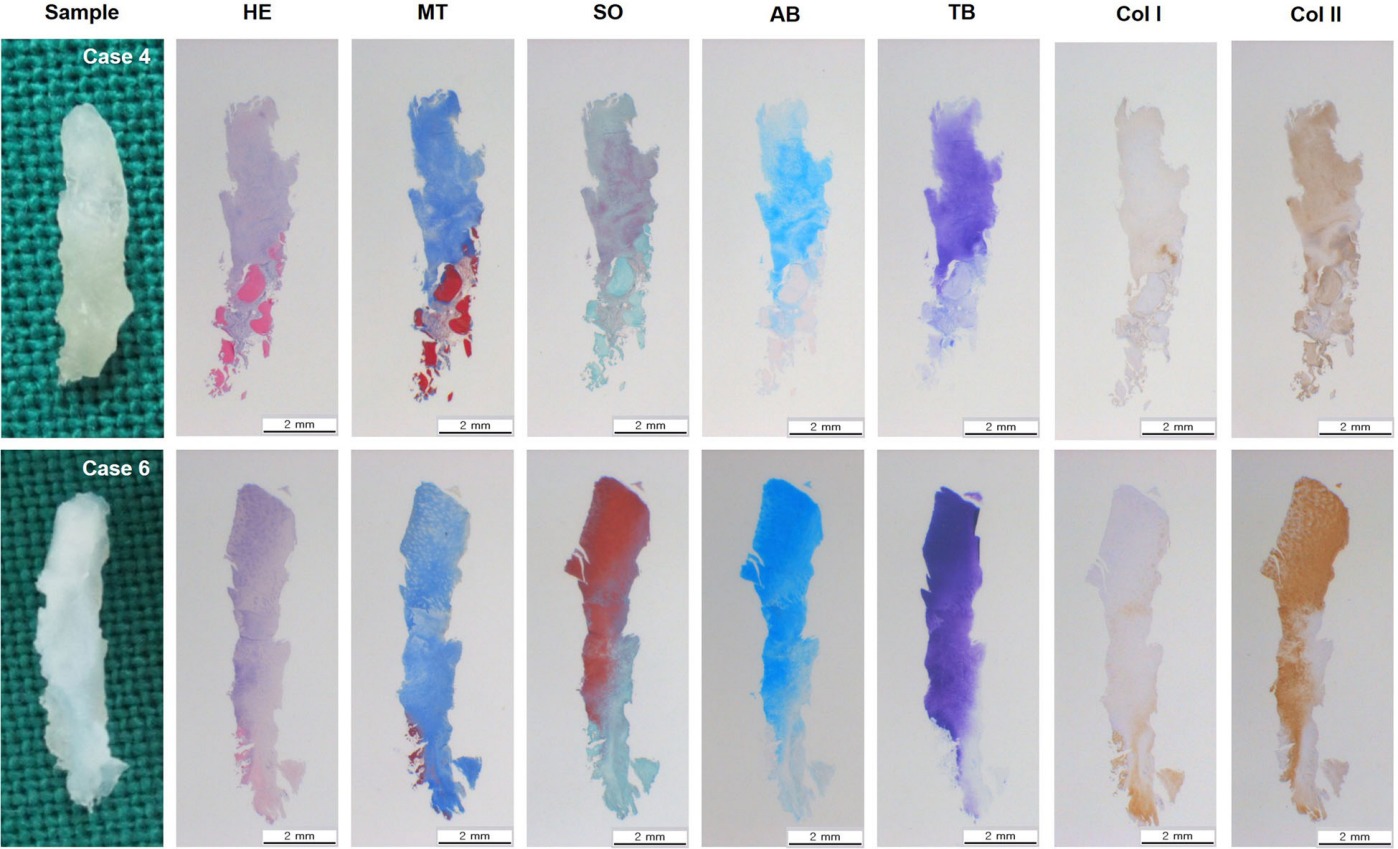
Histological analysis of the second-look biopsy harvested from the patients treated with microfracture with atelocollagen augmentation. The images show that the regenerated cartilages were abundant in type II collagen with hyaline-like appearance. HE = hematoxylin & eosin, MT = Masson’s trichrome, SO = safranin O, AB = Alcian blue, TB = Toluidine blue, Col I=Collagen type I, Col II=Collagen type II

Eight patients (6 in group 1 and 2 in group 2) underwent second-look arthroscopic surgery and the tissue biopsy for histologic evaluation (Fig. 4). The ICRS grades of the regenerated cartilage are presented in Table 5. In the second-look arthroscopy, there was improvement in ICRS grades in all patients. In group 1, two patients improved from grade IV before undergoing arthroscopic microfracture to grade 0 at the 2-year second-look arthroscopy, one patient improved from grade IV to grade I, two patients improved from grade III to grade I, and one patient improved from grade III to grade II. In group 2, two patients improved from grade III to grade I. The histological analysis of the regenerated cartilage in group 1 showed the presence of abundant type II collagen with hyaline-like appearance (Fig. 5). The histological assessment scale is presented in Table 6. Two specimens from group 2 did not show any regenerated cartilage; thus, evaluation could not be performed, and the small number of specimens obtained from both groups did not allow statistical analysis. No study-related adverse events were observed, while six adverse events reported were not related to the current study.

**Table 6:** Histological outcomes of six patients in group 1 (microfracture + atelocollagen augmentation)

Patients	Cartilage thickness	Os score								Immunohistochemistry		
		Tissue morphology	Matrix staining	Surface architecture	Chondrocyte clusters	Mineral	Blood vessel	Basal integration	Total score	Collagen type I	Collagen Type II	Total Score
1	2.51	2.33	0.00	0.67	0.50	0.67	1.00	1.00	6.17	1.00	0.67	1.67
2	4.00	3.00	1.00	2.00	0.83	1.00	1.00	0.00	8.83	1.00	1.00	2.00
3		2.00	0.67	0.00	1.00	0.67	1.00	0.00	5.34	1.00	1.00	2.00
4	4.45	1.33	0.33	0.67	1.00	1.00	1.00	0.67	6.00	0.00	0.33	0.33
5		1.33	0.33	0.33	0.83	1.00	1.00	0.00	4.82	0.33	0.33	0.33
6	6.7	2.00	1.00	1.00	1.00	1.00	1.00	0.67	7.67	1.33	1.33	2.66

*Os score* Oswestry score

## Discussion

The present study’s most important finding was that in patients with OLT, the quality of the regenerated cartilage was superior after arthroscopic microfracture with atelocollagen augmentation than that after arthroscopic microfracture alone. The clinical outcomes assessed 2 years postoperatively were superior in patients who underwent arthroscopic microfracture with atelocollagen augmentation compared to those who underwent arthroscopic microfracture alone, but the differences were not statistically significant. Both groups showed significant improvements in clinical outcomes compared with the preoperative values.

Our hypothesis that the quality of the regenerated cartilage would be superior in patients who underwent arthroscopic microfracture with atelocollagen augmentation compared to those who underwent arthroscopic microfracture alone was confirmed on MRI assessments. The mean MOCART score 2 years postoperatively was significantly superior for group 1 compared to group 2 (67.95 ± 15.61 vs 53.36 ± 12.24, *p* = 0.001). The fibrous cartilage produced by arthroscopic microfracture usually presents inferior biochemical and biological properties compared with the native cartilage; thus, the quality of the regenerated cartilage is important [13]. Once the quality of the regenerated cartilage improves, we believe there may be a greater possibility for maintaining good clinical outcomes in the long term.

Many strategies have been introduced to improve the quality of the regenerated cartilage [28]. Autologous chondrocyte implantation is considered an effective procedure to produce hyaline-like cartilage [29]; however, it is expensive and involves two-stage surgical procedures, with the associated morbidity of harvesting a small portion of the normal articular cartilage [29, 30]. This procedure is often performed with an open approach, which generally requires malleolar osteotomy and extensive arthrotomy that may result in malunion, ankle stiffness, and longer rehabilitation [29, 30]. An autologous matrix-induced chondrogenesis (AMIC) technique involving microfracture and application of a collagen type I/III bilayer matrix has been proposed [31, 32]. Collagen is the connective tissue protein that plays a key role in maintaining tissue morphology and can be used as a scaffold during cartilage regeneration [10]. Implanted exogenous collagen can improve the mechanical stability and durability of the cellular environment and is known to be beneficial for the chondrogenic differentiation and cartilage regeneration [31–35]. The collagen matrix is applied in a solid form and stabilizes and protects the released chondrogenic cells from microfracture [31, 32]. This technique is a cost-effective single-step procedure that avoids donor site morbidity and has good clinical outcomes [31–35]. However, the application of collagen matrix still requires an open approach with malleolar osteotomy and the matrix often needs to be maintained with sutures [31, 32]. The recent development of atelocollagen, a highly purified cell-free type I collagen, has provided a substrate to improve matrix stability and to maintain blood clotting at the defect site [8, 10, 11, 36]. Differently from the AMIC technique, a mixture of atelocollagen and fibrin glue can easily be injected arthroscopically on the cartilage defect, which solidifies after polymerization on the site without the need for open capsulotomy, malleolar osteotomy, or suturing of the scaffold that can injure the surrounding native cartilage.

In an in-vitro study, human bone marrow mesenchymal stem cells and human chondrocytes were seeded on a pre-solid atelocollagen scaffold. Both bone marrow mesenchymal stem cells and human chondrocytes were able to efficiently colonize the whole construct, from the surface to the core [12]. In an animal study of 12 rabbits with full-thickness cartilage defects, microfracture with atelocollagen augmentation resulted in significantly higher histological scores than microfracture alone [8]. The regenerated tissue after microfracture with atelocollagen augmentation was hyaline-like cartilage. The subchondral bone and cartilage were completely regenerated and smoothly attached to the adjacent normal cartilage. Satisfactory clinical outcomes and cartilage regeneration have been observed following arthroscopic microfracture combined with atelocollagen augmentation in patients with cartilage defects [8, 10–13]. Seventeen patients treated with this technique for OLT presented good postoperative clinical outcomes in terms of the 100-mm VAS (18 ± 7.9), AOFAS (88 ± 6.7), and HSS (87 ± 8.7) scores, which were comparable to those in the current study [8]. In a randomized control trial evaluating patients with cartilage defects undergoing high tibial osteotomy, the quality of the regenerated cartilage assessed on biopsy specimens and on postoperative MRI was significantly superior in patients undergoing microfracture with atelocollagen augmentation compared to those undergoing microfracture alone. To the best of our knowledge, the current study is the first randomized control trial to investigate the application of atelocollagen augmentation for OLT involving the largest sample population (31 patients) compared to other case series [8, 12, 13]. The current study demonstrated that the quality of the regenerated cartilage was superior when this technique was applied with arthroscopic microfracture for the treatment of OLT. Although direct comparison may not be feasible, the mean MOCART score (64.49 ± 18.27) of the regenerated cartilage after microfracture with atelocollagen augmentation in the current study was comparable with that reported in similar studies on OLT that used stem cell or solid-type collagen matrix. In a study of 24 patients with a mean age of 46.1 (range, 21–62) years, the mean MOCART score after a mean of 27.1 months after arthroscopic microfracture with stem cell injection was 62.1 ± 21.8 [37]. In a study of 16 patients with a similar mean age of 42.6 (range 14–74) years, the mean MOCART score at 24 months after the AMIC technique was 51.9 ± 11.6 [35]. Considering that these techniques using stem cells or a solid-type collagen matrix require harvesting of stem cells or malleolar osteotomy, an approach generating comparable quality of regenerated cartilage after microfracture combined with atelocollagen augmentation in a single one-step procedure using a minimally invasive arthroscopic technique, which does not require donor site harvesting, would surely be more advantageous. However, comparative studies are required to confirm this hypothesis.

The study is limited by the short follow-up period. The clinical outcomes assessed 2 years postoperatively were superior with an additional atelocollagen augmentation, but the differences were not statistically significant. A longer follow-up would have allowed to observe more mature regenerated tissue, as it is known that the cartilage maturation process can last up to 3 years [38], and demonstrate significant difference in clinical outcomes. Given the fact that the quality of the regenerated cartilage was significantly superior when atelocollagen augmentation was added, we expect its superiority to be maintained in a long-term follow-up. A long-term study of the cohort is required to confirm these findings. Another limitation of the study is the shortage of histological analysis of the regenerated cartilage [39]. Only 8 (18%) patients underwent second-look arthroscopic surgery and the tissue biopsy for histological evaluation. Two specimens from group 2 did not exhibit any regenerated cartilage suitable for histological evaluation. Such a small number of specimens obtained from both groups made it impossible to draw any conclusion. Nonetheless, we thought the available findings would be worth reporting, as there are no histological reports on regenerated cartilage after microfracture with atelocollagen augmentation for OLT. Instead of drawing conclusions from the histologic analysis, a structured morphological assessment was performed using postoperative MRI according to the MOCART score, which is a validated assessment tool with excellent interobserver agreement that is considered a reliable index to evaluate repaired cartilage [13, 25, 26]. The study is slightly underpowered as the power calculation resulted in an estimated sample size of 60 with a 20% drop out rate, when in fact, we had a drop out rate of 25%.

## Conclusion

The quality of the regenerated cartilage was superior after arthroscopic microfracture with atelocollagen augmentation compared to that after microfracture alone in patients with OLT. Significant improvement of clinical outcomes was observed for arthroscopic microfracture with or without an additional atelocollagen augmentation. However, no significant between-group differences were observed in the clinical outcomes.

## Data Availability

The datasets used and analyzed during the current study are available from the corresponding author on reasonable request.

## Abbreviations

OLT: Osteochondral lesion of the talus
VAS: Visual analog scale
HSS: Hannover scoring system
AOFAS: American Orthopedic Foot and Ankle Society
MOCART: Magnetic resonance observation of cartilage repair tissue
MRI: Magnetic resonance imaging
CONSORT: Consolidated Standards of Reporting Trials
ICRS: International Cartilage Repair Society
ITT: Intention-to-treat
PP: Per-protocol
MCID: Minimum clinical important difference
